# Mujeres Unidas: A Pilot Study to Educate Latina Women

**DOI:** 10.1007/s10903-024-01636-z

**Published:** 2024-10-05

**Authors:** Danika Comey, Cassidy Crawford, Isabela Romero, Reyna Sundell, Sophia Thompson Padron, Harley Brittenham, Emily Wiley, Sally Moyce

**Affiliations:** Montana State University, Bozeman, MT USA

**Keywords:** Latina, Immigrants, Anxiety, Mental health

## Abstract

In the United States, it is estimated that 15% of Latinos will experience a depressive or anxiety disorder during their lifetime. Education, prevention programming, and health interventions around topics such as stress, nutrition, mental health, and health maintenance for Latino immigrants are lacking, inadequate, or nonexistent. This type of programming may be protective against depression and anxiety. A total of 19 Latina women completed a five-week, group-based intervention to learn about stress, stress management, nutrition, mental health, and healthy behaviors in a culturally sensitive environment taught by native Spanish speakers. Program evaluation occurred through in-depth interviews and changes in anxiety and depression scores via the Generalized Anxiety Scale and the Patient Health Questionnaire, respectively. The team saw statistically significant decreases in the GAD-7 scores pre- and post-intervention (8.00 versus 5.08, *p*<0.05), but no differences in the PSS or the PHQ-2 scores. Group-based intervention and education taught by native Spanish speakers may be an acceptable and feasible approach to addressing anxiety in Latina immigrants.

## Introduction

Latinos in the United States represent 19% of the U.S. population and a total of 62.1 million individuals [1]. Despite the high proportion of Latinos living in the United States, mental health services offered in Spanish and culturally sensitive care is lacking [2–4]. In many areas, including in the state of Montana, mental illness prevention programming is nonexistent which contributes to health inequities and health disparities between the Latino immigrant community and the non-Hispanic white community [5]. Programs that include education around topics such as stress, nutrition, and mental health maintenance delivered in a group setting may be protective for Latino immigrants and may decrease depression and anxiety symptoms.

It is estimated that 15% of Latinos living in the United States will experience a depressive or anxiety disorder, and an additional 4.4% of Latinos will experience post-traumatic stress disorder (PTSD) [6–8]. An alarming 75% of Latina women report having experienced trauma during their life. Latina women experience higher rates of trauma, including political trauma and sexual trauma, when compared to non-Hispanic whites [9]. Stress, depression, anxiety, and trauma can all impact an individual’s ability and confidence to access healthcare services including mental healthcare. On average, 17% of the non-Hispanic white population will use mental health services in a given year compared to only 7% of Latinos [10]. There is a need for culturally sensitive healthcare and education provided in Spanish to Latino communities around stress, depression, anxiety, and trauma [11].

Preliminary data collected in a rural community in southwest Montana indicates that insurance coverage for Latino immigrants is well below that of non-Hispanic whites. In this region, 86% of Latinos do not have health insurance compared to only 11% of the white population [12, 13]. When compared to those with health insurance, those without health insurance are less likely to receive preventative care, less likely to receive follow-up care, and less likely to fill their prescriptions [14, 15]. The combination of a lack of insurance and insufficient mental health services make accessing care challenging.

To address the increasing need for community services that meet the needs of the growing, underserved Latino immigrant community, a team of researchers developed a curriculum to convene and educate Latina women. The intervention was tailored to Latina women and specifically to mothers because of the influence Latina mothers have on their families’ diets and nutrition [16]. The researchers wanted to capitalize on the role of Latina women in socializing, family health, childhood obesity prevention, and community connectedness so classes were delivered in a group setting [17–19]. The intervention was created with input and expertise from Latina women themselves who were representative of the targeted community.

The aim of this paper is to describe the curriculum and preliminary outcomes of three cohorts of Latina women who completed a group-based educational intervention, which was evaluated using the RE-AIM framework. RE-AIM can be used as an evaluation metric to evaluate the Reach, Effectiveness, Adoption, Implementation, and Maintenance of an intervention. The research team hypothesized that women who participate in a group-based educational intervention would gain knowledge and social support to encourage healthy behavior modifications and improve mental health.

## Methods

### Intervention

The intervention was a five-week, group-based class that met weekly in person in 2022 and 2023. The program was designed by a bilingual, bicultural team of nurses, public health specialists, and master’s students in nutrition and in counseling and modeled after previous nutrition intervention work done by *promotoras* (community health workers) [20]. Based on feedback from community partners and results of previous research, topics were selected to teach stress management, mindfulness, nutrition, and healthy behaviors that are culturally relevant and in Spanish. The program is called *Mujeres Unidas*, United Women.

The educational sessions were taught in Spanish by different specialists within the research team. On the research team, two of the five members were native Spanish speakers. Expertise from leaders who are part of the community can help with program success [21]. One research team member was a counseling graduate school student from Peru with experience facilitating group sessions, another team member was a bilingual graduate research student in the nutrition program, and the final student was a senior nursing student from Puerto Rico in their last semester of university (see Table 1).

**Table 1:** Overview of session content and instructors

	Session Name (Spanish)	Session Name (English)	Instructor
Session #1	Bienvenida y cómo reconocer el estrés en el cuerpo	Welcome and how to recognize stress in the body	Graduate student in counseling from Peru
Session #2	Lectura de etiquetas de alimentos y cómo cocinar bajo en sal	Reading food labels and how to cook with less salt	Bilingual graduate student in nutrition
Session #3	Introducción al cambio de estilo de vida para un cuerpo sano	Introduction to lifestyle change for a healthy body	Nursing student from Puerto Rico
Session #4	Planes alimenticios: DASH y Mediterráneo	Eating plans: DASH and Mediterranean	Bilingual graduate student in nutrition
Session #5	Cómo manejar el estrés	How to manage stress	Graduate student in counseling from Peru

The sessions were hosted at a local non-profit in a central community location. Sessions were conducted in Spanish and most classes were taught by native Spanish speakers who were both bilingual and bicultural. Most study participants were monolingual Spanish speakers. Sessions were held on Monday evenings and lasted 90 min. If a participant had barriers to transportation, one of the research team members would pick the participant up.

### Participants

Participants were a convenience sample recruited from community health screening events, word-of-mouth promotion, and advertisements in a private Spanish-language Facebook group. Following the intervention, each cohort of women were asked to recommend other women who may benefit from the classes. Eligible participants were Spanish-speaking adult women in the local area. Participants were organized into a WhatsApp group message in each cohort to send reminders and announcements about the course. The cohorts had 5–10 women in each and were operated sequentially.

### Study Measures

To determine estimates of acceptability and effectiveness of the intervention to study participants, the evaluation was structured according to parts of the RE-AIM framework [22, 23]. RE-AIM can be used as an evaluation metric to evaluate the Reach, Effectiveness, Adoption, Implementation, and Maintenance of an intervention. While typically used for multi-level interventions, it was selected to evaluate this program to determine whether it could be scaled up and implemented in different settings. Because adoption refers to the implementation of an intervention at a systems or organizational level [22], it was unable to be measured it in this study. Estimated preliminary effectiveness was conducted through measures of mental health and knowledge in a pre- and post-intervention approach.

Prior to beginning the course, participants completed a Spanish version of the Perceived Stress Scale (PSS, α 0.78 Spanish version [24]), the Patient Health Questionnaire (PHQ-2, α 0.67 Spanish version [25]), and the General Anxiety Disorder (GAD-7, α 0.94 Spanish version [26]) electronically on tablets [24, 27]. The PSS is a measure of stress perceived by the participant. It consists of a ten-point questionnaire with scores ranging from 0 to 40. Higher scores on the PSS indicate higher levels of perceived stress [24]. The GAD-7 is a seven-point scale that measures anxiety severity [28]. Results range from 0 to 21 and are categorized into four categories: minimal anxiety, mild anxiety, moderate anxiety, and severe anxiety with higher scores indicating higher levels of anxiety [29]. Finally, the PHQ-2 is a two-point questionnaire that inquires about the frequency of depressive mood over the previous two weeks. Scores for the PHQ-2 range from 0 to 6. If a patient’s score is three or greater, a major depressive disorder is likely [27]. At the end of the final session, participants completed each of these measures again.

The research team estimated the remaining elements in the RE-AIM framework (except Adoption) through semi-structured qualitative interviews one-month following the completion of each cohort. Phone interviews were conducted in Spanish by bilingual research assistants. Interviews lasted between 30 and 45 min. Interviews were audio-recorded, transcribed into English, and checked for accuracy. Transcripts were translated by bilingual research assistants. Sample questions are provided in Table 2.

**Table 2:** Sample questions in English and Spanish from semi-structured interview guide to determine RE-AIM

English	Spanish
**Reach**: How did you hear about Mujeres Unidas? What made you interested in joining? (e.g., what information attracted you to the program) What did you think the program could help you accomplish? What do you think are the best ways to tell people about the program?	**Alcanzar**: ¿Cómo te enteró de la existencia de Mujeres Unidas? ¿Qué te hizo interesarte en unirte? (por ejemplo, qué información te atrajo del programa) ¿Qué pensó que el programa podría ayudarte a conseguir? ¿Cuáles crees que son las mejores formas de informar a la gente sobre el programa?
**Effectiveness**: What are some of the ways Mujeres Unidas has had a positive impact in your life? Please share any ways Mujeres Unidas had a negative impact on your life.	**Eficacia**: ¿Cuáles son algunas de las maneras en que Mujeres Unidas ha tenido un impacto positivo en tu vida? Por favor, comparta cualquier manera Mujeres Unidas tuvo un impacto negativo en su vida.
**Implementation (personal)**: What parts of Mujeres Unidas helped you stay involved and attend all the classes? What things did you look forward to each week? What did you think of the WhatsApp text chain? Did that help you stay engaged? Is there anything we could change to help other women complete all the sessions and attend all the classes?	**Retención**: ¿Qué aspectos de Mujeres Unidas te ayudaron a seguir participando y a asistir a todas las clases? ¿Qué cosas te hacían ilusión cada semana? ¿Qué te pareció la cadena de mensajes de WhatsApp? ¿Te ha ayudado a mantener el interés? ¿Hay algo que podríamos cambiar para ayudar a otras mujeres a completar todas las sesiones y asistir a todas las clases?
**Implementation (setting)**: Mujeres Unidas was delivered in weekly classes on Monday evenings. How do you think the classes should be delivered in the future? How often should the class meet? For how long? In person, via web conference? Where should the class be held?	**Implementación**: Mujeres Unidas se impartió en clases semanales los lunes por la tarde. ¿Cómo crees que deberían impartirse las clases en el futuro? ¿Con qué frecuencia? ¿Durante cuánto tiempo? ¿En persona, por conferencia web? ¿Dónde deberían impartirse las clases?
**Maintenance**: Now that the classes are over, what, if anything, are you doing to maintain your health? What challenges, if any, have you faced with any of these changes?	**Mantenimiento**: Ahora que han terminado las clases, ¿qué hace para mantener tu salud? ¿Qué dificultades, si las hay, has tenido que afrontar con alguno de estos cambios?

All study procedures were reviewed and approved by the Institutional Review Board at Montana State University IRB 2022-265-EXPEDITED in spring 2022. Written consent was obtained by participants at the first class sessions in November 2022, February 2023, and March 2023. To ensure a full understanding of the research and consent process, the research team verbally went over the consent form in Spanish with each group at the start of the program.

### Data Analysis

All data was collected and stored on Smartsheet, which is password protected and HIPAA compliant. The data could only be viewed by the PI and the research lead. Data analysis was conducted on the pooled sample (all three cohorts) using STATA. Researchers analyzed changes in pre- and post-intervention scores for the PSS, the PHQ-2, and the GAD-7 using paired t-tests in STATA.

Interview transcripts were uploaded to Dedoose qualitative data analysis software for thematic coding. A separate team of three research assistants not directly involved in the creation or delivery of the intervention and led by the PI were assembled to code the transcripts. Using thematic analysis, the three members of the analysis team independently coded a random sample of the transcripts to identify initial research themes. The team met and devised a final codebook with themes, sub-themes, and definitions. Themes were categorized based on the RE-AIM framework. All members of the analysis team independently read and coded all interviews, categorizing quotations based on themes and sub-themes in the codebook using Dedoose.

## Results

Demographics for research participants can be found in Table 3. Due to some technological issues with the tablets used in the study, not all participants responded to all participant demographic questions. Most participants were from Mexico, had been in the United States for at least three years, and worked as a housewife.

**Table 3:** Participant demographics (*N* = 19)

Measure	Item	*N* (%)
Age (years old)	< 18 18–25 26–35 36–45 46+ No response	0 (0%) 1 (5%) 7 (37%) 6 (32%) 2 (10%) 3 (16%)
Country of Origin	Colombia Honduras Mexico Venezuela No response	1 (5%) 2 (10%) 11 (59%) 2 (10%) 3 (16%)
How Long in the US (in years)	< 1 1–2 3–5 6–10 11–20 20+ No response	1 (5%) 2 (11%) 4 (21%) 4 (21%) 1 (5%) 3 (16%) 4 (21%)
Occupation	Childcare Provider Housewife Volunteer Food Service Other No response	1 (5%) 7 (37%) 1 (5%) 3 (16%) 4 (21%) 3 (16%)
Marital Status	Single Married Divorced Other No response	0 (0%) 13 (68%) 2 (11%) 1 (5%) 3 (16%)
How Many Children	0 1 2 3 4 5+ No response	2 (10%) 3 (16%) 4 (21%) 3 (16%) 3 (16%) 0 (0%) 4 (21%)
How Many Children Living in the Home	0 1 2 3 4 5+ No response	5 (26%) 2 (11%) 4 (21%) 2 (11%) 2 (10%) 1 (5%) 3 (16%)

### Reach

Reach refers to the number of individuals who engage in the intervention. Information was not collected about the number of persons the recruitment messages reached; however, the research team recruited 10 participants in each cohort. In Cohort 1, one participant only attended the first session before dropping out, leaving nine total participants. In Cohorts 2 and 3, five women completed the entire intervention. The final sample for all three cohorts was 19 women total.

Interview data reveal that the women were interested in the program to learn new information and to connect to other women. One participant said,

You are not the only one who has problems, who goes through things. There are many, many women who are in that situation. (Cohort 1)

### Preliminary Effectiveness

Preliminary effectiveness estimates the impact of the intervention on targeted outcomes. Because of the small sample size, the research team was unable to estimate true effectiveness of the intervention but saw statistically significant differences in the GAD-7 scores pre- and post-intervention (pre-scores mean 8.00, post-scores mean 5.08, *p* < 0.05), but did not see statistically significant changes in the PSS or the PHQ-2. Full results are shown in Table 4. Women who scored three or more on the PHQ-2 were referred for further mental health evaluation and support at a local community clinic affiliated with the University after the completion of the intervention.

**Table 4:** Results of stress, depression, and anxiety tests

	Pre/Post Test				
	Pre-Test	Post-Test	*t*	*df*	*p-value*
PSS	31.5000 (4.9818)	32.8333 (4.3866)	-1.0086	11	0.3348
PHQ-2	2.7500 (1.9129)	1.8333 (1.0299)	1.8941	11	0.0848
GAD-7	8.0000 (4.0899)	5.0833 (2.4293)	3.1535	11	0.0092*

Perceived Stress Scale (PSS)

Patient Health Questionnaire (PHQ-2)

Generalized Anxiety Disorder Scale (GAD-7)

Note. * = *p* < 0.05, Standard deviations appear in parentheses below means

### Adoption

Within the RE-AIM framework, adoption refers to a system adopting an intervention, so the research team was unable to measure adoption in this study.

### Implementation

This work conceptualized implementation at both the personal and the setting level. Personal implementation refers to the participants’ use of the intervention; and setting implementation was measured by asking about the participants’ opinions of how the program was delivered.

Women self-reported that the social aspect helped keep them engaged in the program on the individual level. One said,

It was like, it’s Monday I’m going to see my girlfriends and chat to my heart’s content. It’s like that. That’s what it meant for me to use the outlet there. I’m going to be able to talk about this or let off steam. (Cohort 1)

Another said,

I felt confident, I felt good with everyone in the group. Because not only one of them had a problem, but all of us had a problem, a situation, something, a reason to keep coming to the program. I’m going to see my friends again and I’m going to feel good because it’s a time for me alone. (Cohort 2)

The connection the women felt to one another was particularly salient given their immigration experiences. Multiple women commented on the loneliness they feel in the United States and identified Mujeres Unidas as helping to alleviate that:

Like, I was like in a moment like relaxing. It reminded me of when I lived in my country and saying like I’m going to see my friends to get out of the routine and so on. (Cohort 3)

At the setting level, all the women liked the length of the classes and that they were in person. They also appreciated the WhatsApp reminder texts. They mentioned they would like to have input on the day and time of the intervention; some even wanted to meet multiple times a week. They noted that helping women with transportation would increase participation in future groups.

### Maintenance

Maintenance refers to the lasting effects of an intervention. The team measured effects one-month following completion of the program, and women said they were making lifestyle modifications based on what they had learned in the classes. These effects came in dietary changes, specifically in the amount of salt they use:

I keep going, I exercise. I eat healthier, I don’t eat so much salt. I’m practicing everything I learned in the class. (Cohort 1)

They also noted that they were able to control stress better after participating in the program:

There is something I learned: that stress is something that is part of our life, so it’s something inevitable. We can’t do anything about it. Just try to cope with it the best way we can. I’m a person who stresses a lot. So I try to say no, this is just part of life. Let things flow, let things go and not get so stressed out. I try to apply what I was told. (Cohort 3)

One woman noted that she had a new focus on her own personal health and was investing in herself after the classes:

I put in more effort for my health now. I am eating healthier. I am now putting more effort in myself. (Cohort 2)

Finally, women noted that the social connections they began in the program continued when they were no longer regularly meeting:

There are times when I want to invite [the other women] to have a coffee because they are now my friends and I know it would be no problem if I need someone to talk to so I invited them for coffee. (Cohort 1)

Upon completion of the program, all participants were provided with documents for community resources including the local federally qualified health center, the mental health clinic affiliated with the University, and the local health system. The WhatsApp text message group continues to operate after the intervention and participants can reach out to the research team if they have any questions or concerns about what they learned in the Mujeres Unidas class. This WhatsApp message also allows for the research team to communicate additional opportunities for health events in the area that are specifically targeted for the Latino population including community health fairs, vaccination clinics, and opportunities for community education.

When participants were asked about any challenges they encountered during the course, many noted that the weather made it difficult for them to come to the sessions. Some noted that the sessions were held too late in the evenings for them, and some noted that they wished we were able to provide childcare.

## Discussion

This is a report on a five-week group intervention designed to address stress, nutrition, mental health, and health maintenance among a group of Latina immigrants in the intermountain West. Previous research indicates that group interventions for Latino women can be protective and effective, but little literature exists on the use of culturally sensitive group programming for mental health services for Latina women [30, 31]. Shattell and colleagues conducted a study examining depressed Mexican women [30], but this research expands on this existing work by adding the GAD-7 and opening the study to include participants from additional countries in Latin America beyond just those from Mexico. Other research indicates that Latina women are open and accepting of group interventions, but few studies have actually been designed and implemented to measure the feasibility and acceptability of these interventions [32–34].

Anxiety and depression rates are high among Latina immigrants when compared to non-Hispanic whites [35–38]. Latina women in the United States face challenges such as social isolation, othering, fear, and discrimination [37, 39]. Friendships, social connectedness, and acceptance are all protective factors that impact Latina’s health and wellbeing including mental health [40]. Group therapy among Latina women with content that is culturally sensitive may ease symptoms of anxiety. Not only are there challenges navigating systems that are not set up for immigrants, but there are also constant reminders from the community that they are different from the majority, white non-Hispanic population that may cause stress and anxiety [41]. The lack of Spanish-language services for mental health in a rural area may prevent many from seeking care and require innovative solutions to addressing current need [13, 42]. This intervention may be a creative way to use existing resources to build on the power of social ties.

These results suggest that a female-focused group intervention may be one way to provide women with health education while improving social connection and health behaviors [31]. For Latina immigrant women in particular, finding social connection can be a challenge and may contribute to feelings of isolation and poor mental health including anxiety and depression [43]. This research found that the women in the sample were motivated by the friendships they made in the groups and that those friendships lasted after the groups no longer met. These relationships may be protective against the symptoms of anxiety. Addressing social isolation through group interventions may mitigate some of the depression and anxiety symptoms among Latina women [44, 45]. The new friendships that the women built, the trust they had in their peers and their group members, and their desire to learn about health topics like nutrition created an environment where women felt safe and secure to share their hardships and challenges they faced in the United States as Latina immigrant women. This study is unique as it uses part of the RE-AIM framework and provides culturally sensitive content that was created in partnership with Latina women. This intervention shows that these classes are feasible to implement, acceptable to participants, and had moderate preliminary effects on women’s anxiety levels, as measured by the GAD-7.

The main limitation to this study was the small sample size. Although 19 women completed the intervention as part of the pilot study, it may be difficult to extrapolate the results from this study to a larger sample. Due to a lack of power, the research team were unable to show efficacy of the intervention on mental health outcomes. Long-term implementation of the intervention may be limited due to the study design of interviewing women one-month post-sessions. Finally, while the quantitative effects on mental health outcomes were not particularly robust in this study, the research team believe that the program had a positive effect on mental health based on the qualitative data collected.

## Conclusion

The intervention presented in this article sought to provide health education to Latina women to improve nutrition, health education, and overall mental health. Mujeres Unidas had success in engaging women and in reported markers of behavior change after one month. Future research should focus on expanding group-based interventions to capitalize on the cultural importance of social connection among Latina women [44]. Creative solutions to address provider shortages and a lack of culturally relevant care may provide particular benefits to Latina immigrants who are struggling with anxiety, depression, and social isolation [21]. This pilot study demonstrates that Mujeres Unidas may be a feasible resource for Latina women.
